# An Ultrasonically Powered Implantable Microprobe for Electrolytic Ablation

**DOI:** 10.1038/s41598-020-58090-8

**Published:** 2020-01-30

**Authors:** A. Kim, S. K. Lee, T. Parupudi, R. Rahimi, S. H. Song, M. C. Park, S. Islam, J. Zhou, A. K. Majumdar, J. S. Park, J. M. Yoo, B. Ziaie

**Affiliations:** 1 Department of Electrical and Computer Engineering, Temple University, Philadelphia, PA 19122 USA; 2 Jubilee Biotechnology LLC, Philadelphia, PA 19122 USA; 3 School of Electrical and Computer Engineering, Purdue University, West Lafayette, IN 47907 USA; 4 Birck Nanotechnology Center, West Lafayette, IN 47907 USA; 5 Department of Electronic Engineering, Sookmyung Women’s University, Seoul, South Korea; 6 Pancreatobiliary Cancer Clinic, Department of Surgery, College of Medicine, Gangnam Severance Hospital, Yonsei University, Seoul, South Korea; 7 Department of Microbiology, School of Medicine, CHA University, Seongnam, South Korea

**Keywords:** Targeted therapies, Biomedical engineering

## Abstract

Electrolytic ablation (EA) is a promising nonthermal tumor ablation technique that destroys malignant cells through induction of a locoregional pH change. EA is typically performed by inserting needle electrodes inside the tumor followed by application of direct current (DC), thus inducing electrolysis and creating localized pH changes around the electrodes. In this paper, we report an ultrasonically powered implantable EA microprobe that may increase the clinical relevance of EA by allowing wireless control over device operation (capability to remotely turn the device on and off) and providing flexibility in treatment options (easier to administer fractionated doses over a longer period). The wireless EA microprobe consists of a millimeter-sized piezoelectric ultrasonic receiver, a rectifier circuit, and a pair of platinum electrodes (overall size is 9 × 3 × 2 mm^3^). Once implanted through a minimally invasive procedure, the microprobe can stay within a solid tumor and be repeatedly used as needed. Ultrasonic power allows for efficient power delivery to mm-scale devices implanted deep within soft tissues of the body. The microprobe is capable of producing a direct current of 90 µA at a voltage of 5 V across the electrodes under low-intensity ultrasound (~200 mW/cm^2^). The DC power creates acidic (pH < 2) and alkaline (pH > 12.9) regions around the anode and the cathode, respectively. The pH change, measured using tissue-mimicking agarose gel, extends to 0.8 cm^3^ in volume within an hour at an expansion rate of 0.5 mm^3^/min. The microprobe-mediated EA ablative capability is demonstrated *in vitro* in cancer cells and *ex vivo* in mouse liver.

## Introduction

Surgical resection remains the first therapeutic option for early-stage solid tumors. In advanced metastatic situations, systemic chemotherapy or radiation therapy is typically used to control disease progression. Local thermal ablation using insertable RF or microwave probes has also been used to destroy solid tumors in cases where the tumor is deemed to be nonresectable (e.g., liver or pancreatic tumors close to a sensitive anatomical structure)^1^. Although clinically accepted, thermal ablation methods face limitations in cases where the tumor is located near a major blood vein, for which collateral thermal damage is unacceptable^1–3^. Other ablation methods using laser sources, high-intensity focused ultrasound (HIFU), and cold (cryo-ablation) have also been proposed and are currently at various stages of development and clinical trials^4–6^. Electrolytic ablation (EA), a nonthermal method in which a localized pH region is created via two electrodes inserted into a tumor and connected to a direct current (DC) source, is an attractive method that can controllably treat the tumor and limit the damage to adjacent critical structures (due to the use of nonextreme energy)^2,7–9^. EA was first used to treat lung tumors^10^ followed more recently by its application to liver^7^ and pancreatic tumors^8^. Both thermal and electrolytic methods require the insertion of probes into the tumor under image-guided procedures. Although repeated therapy sessions are preferred and common due to the strongly resistant nature of tumors, each session of cancer treatment, such as chemo- or radiotherapy is usually administered through difficult and cumbersome in-patient procedures^11–14^.

**Figure 1:**
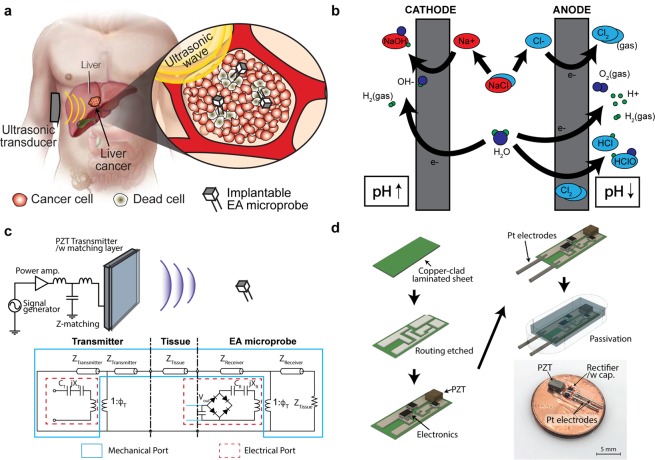
(**a**) Schematic view of wireless electrolytic ablation therapy. Multiple EA microprobes can be implanted inside a tumor and powered remotely by an ultrasonic wave. (**b**) Essential chemical reactions occurring during EA: anode: Cl_2_ (gas), H_2_ (gas), O_2_ (gas), HCl, HClO; cathode: NaOH, OH^−^, H_2_ (gas). (**c**) Ultrasonic power: schematics of the power transfer link and its theoretical electromechanical model^38^. (**d**) Fabrication process: 1. circuit is drawn on copper-clad laminated polyimide sheet (Pyralux), 2. circuit layout lithography and etching, 3. soldering components, 4. platinum electrode assembly, 5. passivation and optional packaging, and 6. a photograph of the mm-scale electrolytic-ablation device (9 mm × 5 mm × 2 mm).

In this paper, we present an ultrasonically powered implantable microprobe that enables wireless EA therapy. Figure 1a illustrates a schematic of the microprobe implanted in a tumor using a procedure similar to the deployment of brachytherapy seeds. The device is powered from outside of the body using ultrasound and subsequently delivers direct current to the tumor, inducing an *in situ* local pH change. Ultrasonic power allows the device to be miniaturized to dimensions (mm-scale) that are not feasible using traditional inductive wireless powering methods^15^. In addition, ultrasound has a long operation range (>10 cm)^15–20^ and a higher power transfer efficiency than traditional inductive power at mm-scale reviver sizes^15^, and it is significantly less sensitive to angular misalignment between the transmitter and the receiver^15,17,20–23^. The implantable microprobe may increase the clinical relevance of EA by allowing wireless control over device operation (capability to remotely turn the device on and off) and providing flexibility in treatment options (easier to administer fractionated doses over a longer period). Although the described microprobe requires initial insertion using a biopsy needle or a trocar, once in place, it can be remotely reactivated if further treatments are necessary without repeated insertion of needle electrodes.

## Results and Discussion

### Electrolytic ablation principles

Figure 1b illustrates the essential electrochemical reactions during *in situ* local pH modulation with low-voltage DC-induced electrolysis. The primary effect of electrolysis is the generation of hydrogen (hydronium) and hydroxide ions (anode: 2*H*_2_*O ↔ O*_2_ + *4H*^+^ + *4e*^−^ and 2*Cl*^-^ → *Cl*_2_ + *2e*^−^; cathode: *2H*_*2*_*O* + *2e*^−^ *↔ H*_*2*_ + *2OH*^−^)^8^. A secondary effect of electrolysis involves sodium chloride (*NaCl*), which forms *Na*^+^ ions that move toward the cathode and *Cl*^−^ ions that move toward the anode. These ions react with the aqueous *in vivo* environment to produce sodium hydroxide and hydrogen at the cathode (2*NaCl* + 2*H*_2_*O ↔ Cl*_2_ + *H*_2_ + 2*NaOH*) and hydrochloric acid, oxygen, and chlorine at the anode (*2Cl*^−^ *↔ Cl*_*2*_ + *2e*^−^ and *Cl*_*2*_ + *H*_*2*_*O ↔ HClO* + *H*^+^ + *Cl*^−^*)*. As a result of reactions, the region surrounding the anode becomes acidic (pH < 6), whereas the region around the cathode becomes alkaline (pH > 9). This localized pH change can cause cell death around the electrodes.

### Wireless ultrasonic powering

An ultrasonic power link consists of three elements: an ultrasonic transmitter, soft tissue, and a receiver (microprobe in our case) (Fig. 1c-top). The microprobe converts incoming ultrasonic waves into a direct electrical current and induces electrolysis through a pair of platinum electrodes in the tumor. (Fig. 1c-bottom) illustrates the theoretical ultrasonic power schematic equivalent circuit using the Krimholtz, Leedom, Mattaei (KLM) model (detailed theoretical discussion of the KLM model is provided in the Supplementary Information)^24^.

**Figure 2:**
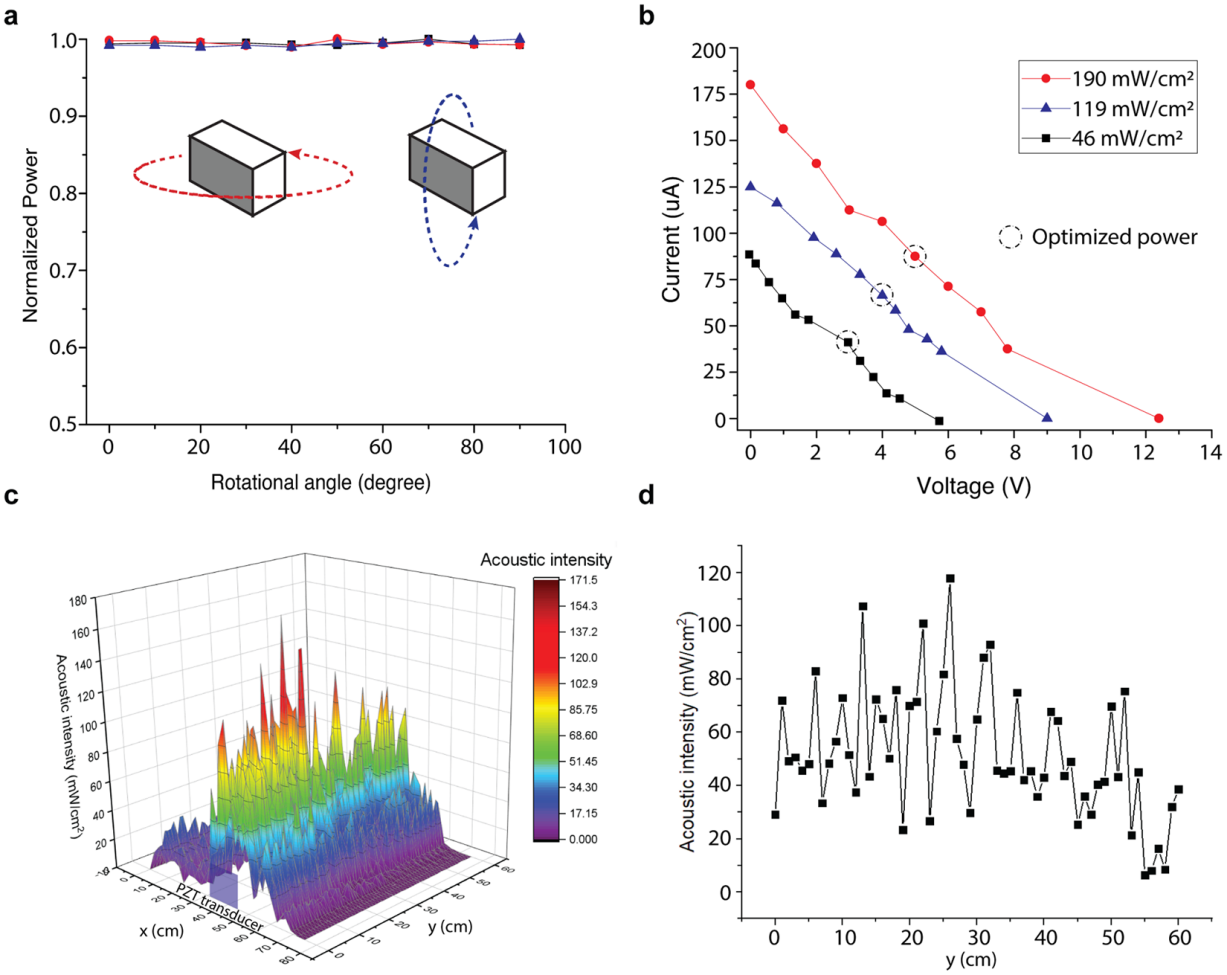
Wireless ultrasonic power: (**a**). Transverse and axial rotational angular misalignment, (**b)**. I–V characteristics at 190 mW/cm^2^ ultrasonic intensity, (**c**). Nonreflected ultrasound propagation across the frontal region of the ultrasonic transducer, (**d**) Distribution of ultrasonic intensity seen on the y-axis.

Prior to testing the efficacy of the microprobe, we investigated ultrasonic power for the intended microprobe form factor (the receiver size of 2 × 4 × 2 mm^3^; see Methods section for detail) (Fig. 1d). Ultrasonic power for the microprobe was characterized in terms of angular alignment sensitivity between a transmitter and a receiver, current-voltage (I–V) characteristics, and the field of operation. Figure 2a shows the normalized received electrical power as a function of transverse and axial rotational angular misalignment measured under open-circuit conditions. The received output remained constant over 90° rotations, confirming the misalignment insensitivity^15^. The I-V characteristics of the microprobe were also measured to estimate the output power at three different ultrasonic intensities: 46, 119, and 190 mW/cm^2^, corresponding to mild, moderate and strong ultrasonic intensities, respectively (Fig. 2b). The electrical power from the microprobe with platinum electrodes was measured to be 164 μW (V = 3.5 V, I = 47 μA), 300 µW (V = 4.23 V, I = 71 µA), and 450 µW (V = 5 V, I = 90 µA) under mild, moderate, and strong ultrasonic intensities, respectively. These results confirmed that sufficient power can be supplied to initiate electrolysis (the wireless power transfer efficiencies were 2.7%, $\eta =|{P}_{output}/{P}_{input}|$)^23^.

The field of operation, a critical factor that determines the ablation zone and the efficacy of the treatment, was also evaluated. We measured the acoustic intensities in an arbitrary volume (60 cm^3^) in front of the transmitter with a 1-mm spatial resolution. We used a fiber optic hydrophone (FOH, Precision Acoustics, UK)^25^ mounted on a manipulator (XY plotter, MakeBlock Ltd.) while manually changing the Z location to characterize the field of operation under nonreflective boundary conditions by covering the water tank walls with acoustic absorption layers (Aptflex F28, Precision Acoustics, UK). Figure 2c shows a three-dimensional map of acoustic intensity, and Fig. 2d shows the distribution of acoustic intensity along the traveling direction (y-axis). Overall, ultrasound intensities of over 60 mW/cm^2^ (average) were measured for distances of up to 60 cm away from the transducer. Additionally, the regional peak intensities tend to concentrate along the center axis of the transducer (with a measured intensity of 120 mW/cm^2^ at a distance of 25 cm from the transducer). Such a long working distance is sufficient for most deeply seated solid tumors^20,26^.

### Ablation zone estimation

**Figure 3:**
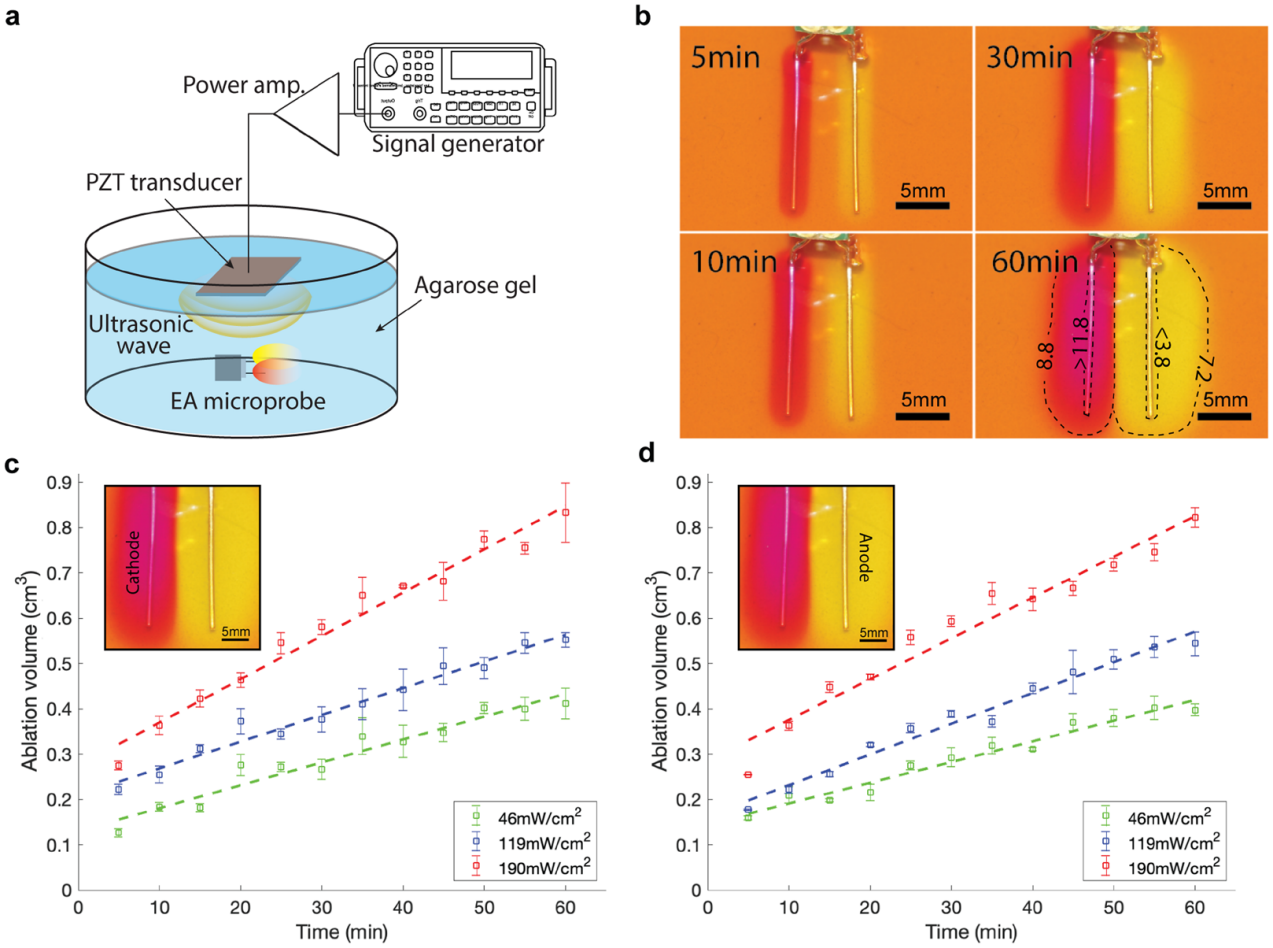
(**a**) Experimental setup used to record the *in vitro* ablation zone. An EA microprobe was placed in agarose gel loaded with a pH-sensitive dye and powered by an ultrasonic transducer (figure is not to scale). **(b**) Time-lapse photographs showing ablation zone expansion around the cathode (left) and the anode (right). Measured pH values marked at concentric points around the electrodes. All scale bars: 5 mm. (**c**) Progression of the area of the ablation zone (pH fronts) as a function of time around the cathode and (**d**). Anode at three different ultrasonic intensities: 46, 119, and 190 mW/cm^2^. Error bars represent SD.

Using a tissue-mimicking agarose gel with a pH indicator (phenol red)^27^, we showed that the ablation zone (treatment locality) can be easily controlled by tuning the treatment time and the input ultrasonic power. The ablation zone (evaluated by measuring the pH front) as a function of time is shown in Figure 3c,d. The ablation zone was estimated by measuring the area of color change by phenol red dye, red near the cathode and yellow near the anode. We observed isotropic expansion of the ablation zone at a constant rate for a given ultrasonic intensity over time. The ablation zones after 30 min of operation for the anode/cathode were 0.25, 0.35, and 0.6 cm^3^ for ultrasonic intensities of 46, 119, and 190 mW/cm^2^, respectively. The ablation zone volume expanded linearly from the anode/cathode as a function of time. The average rates of ablation zone expansion were respectively 0.4/0.5, 0.7/0.6, and 0.9/0.9 mm^3^/min under mild, moderate, and strong ultrasonic intensities. The pH front was simultaneously measured using an electrode-type pH meter (IC-401, Specialty Sensors LLC) in a 20 G needle. The measured pH was as low as 1.7 to 3.8 near the anode and as high as 11.8 to 12.3 near the cathode (average pH diffusion rate was 0.09/min) (Fig. 3b).

The ablation zone expansion size and rate are a function of ionic mobility and the applied electric field (similar to the electrophoresis). Although the pH-indicated ablation zone is only an estimation and may not perfectly correspond with the distribution of cell death around the margin, it is generally agreed that EA can be designed to tailor an ablation zone based on pH fronts and tissue electric resistance (also related to ionic mobility). The appropriate treatment scenario must still be elucidated in animal models (e.g., using mild ultrasound with short duration to treat a region requiring extreme locality or a long single session with strong ultrasound to cover a larger volume).

### *In vitro* validation

**Figure 4:**
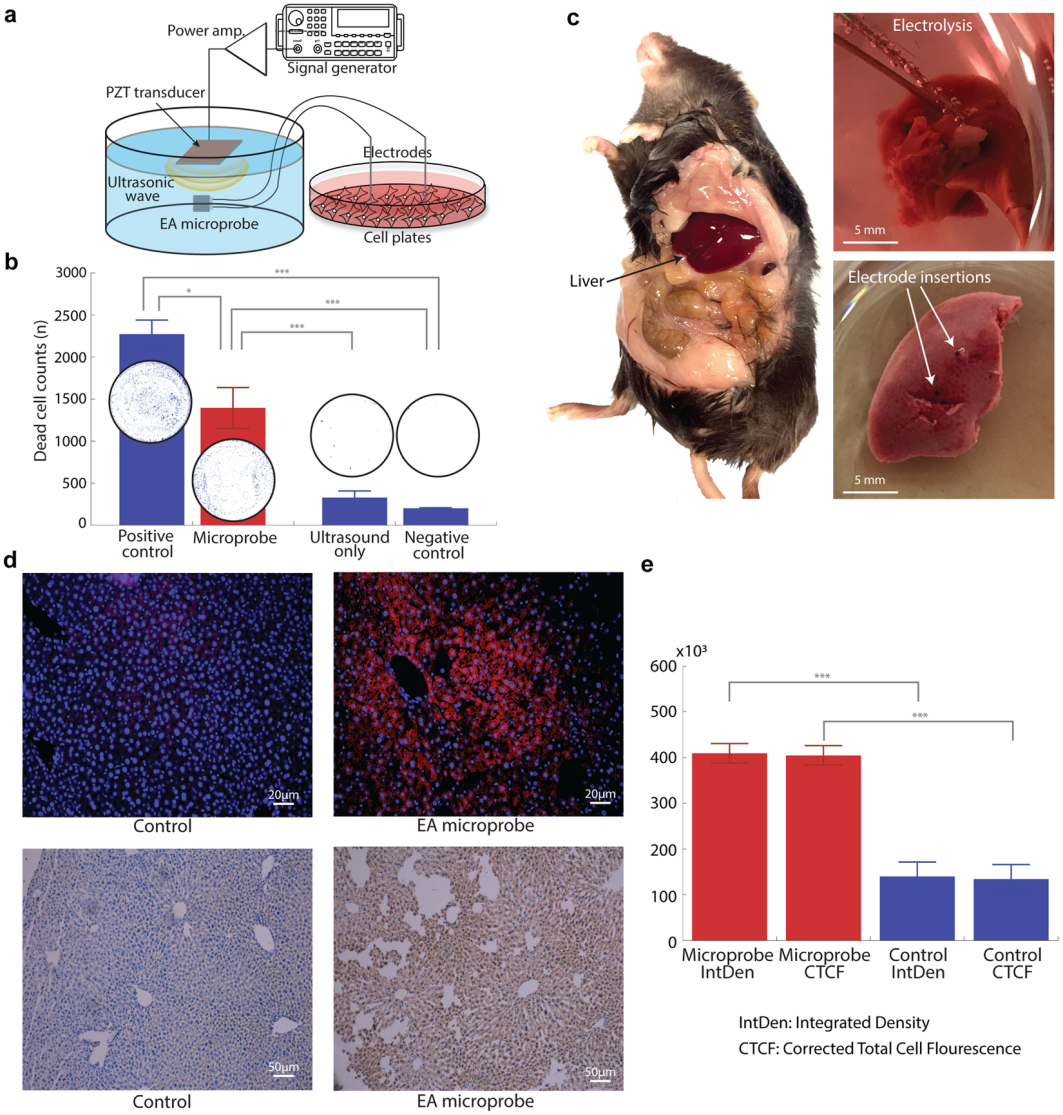
The biological action of EA microprobe: (**a**). Experimental setup for *in vitro* cell viability (figure is not to scale), (**b**). Cell viability assay: The microprobe is effective as a DC power source (positive control) compared to negative controls (ultrasound only and no action). (**c**) *Ex vivo* experiment using mouse liver. (**d**) The expression of caspase3 was detected by immunofluorescence and immunohistochemistry. Positive caspase3 is counterstained with DAP in immunofluorescence and hematoxylin in immunohistochemistry. The control group (electrode insertion only) was compared with the experimental group. (**e**) Analysis of immunofluorescence: integrated density and corrected cell fluorescence were measured. Error bars represent SD; n ≥ 4; p-value was determined using a two-tailed t-test. *p < 0.05, **p < 0.01, ***p < 0.001.

We evaluated the efficacy of the microprobe-mediated EA with an *in vitro* cell viability assay by inducing electrolysis in HMT3522 S1 breast cancer cell cultures (Fig. 4a). We used four experimental groups: (1) the microprobe group, (2) the positive control (i.e., electrolysis using a laboratory DC power source), (3) the negative control (i.e., no action), and (4) the ultrasound-only group. Each group had three cell wells, and all the experiments were repeated at least quadruple (n ≥ 4). The microprobe group showed significant cell death, comparable to the positive group (p-value = 0.5254) after 30 min of operation (Fig. 4b). Cell death caused by the microprobe was statistically significant compared to the negative control group and ultrasound-only group (the p-value was less than 0.0001 for both). The average number of dead cells was 1,394 spots (± 241 spots) in the microprobe group, 2,272 spots (± 166 spots) in the positive control group, 327 spots (± 78 spots) in the ultrasound-only group, and 201 spots (± 5 spots) in the negative control group. Figure 4b circular subsets are pictures of wells of the cell plate after Trypan blue staining.

The difference between the microprobe group and the positive control was attributed to the difference in the electrical current; the capacitive source impedance of the microprobe resulted in a lower current than the DC power supply (105 µA at 6.2 V from the microprobe as opposed to 1.02 mA at 6.2 V from the DC power supply, Section B). As such, the strong ultrasonic intensity (190 mW/cm^2^) induced a direct current of 105 µA, which was slightly higher than that measured in the ablation zone experiment (90 µA, Section C) due to the media difference (cell media vs. agarose). Last, the temperature was simultaneously measured throughout the experiments (infrared thermometer, Traceable^®^ Products). We did not observe a significant temperature change (ΔT was less than 1 °C), affirming the EA-induced cell death through the tumor microenvironment alteration^2,9,28,29^.

### *Ex vivo* validation

The *ex vivo* efficacy of microprobe-mediated EA was tested on fresh livers harvested from 6-month-old mice. Unambiguously demonstrated cell death is summarized in Fig. 4c. A total of two groups were prepared: a control group and a microprobe group. The control group received electrode insertion without the operation, whereas the microprobe group received strong ultrasonic intensity (190 mW/cm^2^) for 30 min. Each group had three liver samples, and all the experiments were repeated at least quadruple (n ≥ 4). Compared to the control group, the microprobe group showed a significant increase in cell death. Figure 4d shows images of caspase3 expression in the liver using immunofluorescence and immunohistochemistry. Positive caspase3 was counterstained with DAPI in immunofluorescence and hematoxylin in immunohistochemistry. Total cell death was analyzed using integrated density (the product of area and mean gray value) and corrected total cell fluorescence (CTCF) (by compensating for the background) (Fig. 4e), which were statistically significant compared to the control (p-value = 0.0001); CTCFs were 409 × 10^3^ (± 139 × 10^3^) and 21 × 10^3^ (± 31 × 10^3^) for microprobe and control groups, respectively. Note that the output voltage from the microprobe was 6.20 V (105 µA), which was identical to those in the *in vitro* cell viability experiment.

While the *ex vivo* results confirmed the effectiveness of the microprobe-mediated EA, this study has limitations. The extrapolation of this study to a large animal or human may be difficult because this study mainly utilized *in vitro* cancer cells and mouse liver. The ablation parameters (i.e., ablation zone, treatment time, ultrasonic intensity, delivered DC output, and possible chlorine gas distribution) described herein may not be the same in a large animal or human because the larger volume of body fluid may mitigate the diffusion of pH and reduce (or promote) the ablation zone. Notwithstanding, we expect that the wireless controllability offered by the microprobe can be exploited to optimize the treatment for larger subjects. For example, we expect that implanting multiple microprobes can reduce the treatment time; four to five microprobes can effectively ablate a 4-cm diameter liver tumor with an hour-long single session.

Last, we should emphasize that the microprobe requires further development and validation. Despite its small dimensions, implanted microprobes in the body should be carefully examined to evaluate their biocompatibility, long-term reliability, and efficacy. Additionally, the system may require real-time monitoring, which can be accomplished by a closed-loop system that incorporates telemetry function within the same implants^30^. Alternatively, one can utilize MRI to monitor the pH change in real-time, as demonstrated by Meir *et al*.^31^.

## Methods

### Fabrication of the microprobe

Figure 1d shows the fabrication procedure and the microprobe prototype. Table 1 summarizes the mechanical and electrical properties of the subcomponents of the microprobe. The microprobe is comprised of a mm-scale piezoelectric receiver element (PZT-5A, PSI-5A4E, Piezo Systems Inc., USA), a full bridge rectifier created by four surface-mount Schottky diodes (CMRSH-4DO, Central Semiconductor Corp. USA), a smoothing capacitor (0.1 µF, 1005 × 8R1E103K050BA, TDK), and a pair of platinum electrodes (platinum-clad niobium electrodes, 0.5 mm diameter × 10 mm length, Anomet Products, USA). The entire electronic is housed on a copper-clad polyimide sheet (Pyralux AP9141R, 100 µm polymer thickness, 35 µm copper thickness, DuPont, USA). The circuitry layout was fabricated using lithography followed by etching (Fig. 1d, steps 1-2). A 2 mm-thick PZT-5A plate was pre-diced to 2 × 4 × 2 mm^3^ elements using a dicing saw system (DAD-2H/6 Dicing Saw, Disco, Japan). Next, a PZT slab, a full-wave bridge rectifier, and a smoothing capacitor were substantially assembled (Fig. 1d step 3). Note that a rectifier circuit converts the PZT receiver’s alternating current (AC) into direct current (DC). Then, platinum electrodes were assembled (Fig. 1d, step 4). Finally, the fully assembled microprobe was coated with 5 µm Parylene-C for passivation and biocompatibility (Fig. 1d, step 5). An optional custom packaging can also be added in this step. Figure 1d (bottom right) shows a picture of the microprobe prototype. The overall device dimension was 9 × 5 × 2 mm^3^ with 5 mm active platinum electrode tips for ablation (weight 200 mg).

**Table 1:** Characteristics of the components used in the microprobe.

Property	Value
Longitudinal acoustic wave velocity in PZT-5A	4,350 m/s
Longitudinal acoustic wave velocity in tissue	1,540 m/s
Dimension of the transmitter/receiver PZT-5A	2 × 4 × 2 mm^3^
Resonant frequency (measured)	650 kHz
PZT dielectric constant	1,800
Electromechanical coefficient (*k*^2^)	0.72
Tissue attenuation coefficient	~2.5 Np/cm^39^

### Ultrasonic power

For maximum wireless power transfer efficiency, operation must occur at the resonant frequency (*fr*). This frequency is usually determined by physical properties, size, and aspect ratio of the piezoelectric transducers. In the 1D model, a piezoelectric transducer operates in the thickness-expansion mode, whose resonant frequency is defined by its thickness (*t*) and wavelength (${f}_{r}={v}_{D}/2\times t$, where ${v}_{D}$ is the speed of sound in the piezoelectric material). However, this is true only if the aspect ratio (width/thickness) is greater than 10. In a cuboidal form factor, such as a receiver used in the microprobe (2 × 4 × 2 mm^3^), the resonant frequency shifts due to Poisson’s ratio and the associated mode coupling^19,32,33^. Therefore, the resonant frequency of a PZT slab was measured by an impedance analyzer (Keysight E4990A, CA, USA) over a range of frequencies. The resonant frequency (*fr*) was the point where the internal impedance was the lowest and anti-resonance (*fa*) was the frequency where the internal impedance was the highest. The measured impedance is plotted in Supplementary Fig. 1. Several radial resonant modes were observed at lower frequencies, and the first thickness-mode resonance appeared at a frequency of 650 kHz.

All experiments were conducted in a water tank because water is a common model for soft tissue, as their acoustic impedance are similar (*Z*_*water*_ = 1.48 MRayl, *Z*_*tissue*_, _*avg*_ = 1.63 MRayl)^34^. A microprobe was submerged in a water tank (dimensions of 60 × 30 × 40 cm^3^) and positioned within the near-field range. Figure 1c (top) shows a block diagram of the ultrasonic power, which has three parts: an ultrasonic transmitter, soft tissue, and a microprobe. The left diagram indicates an external ultrasonic power system that consists of a signal generator (MSOX3024T, Keysight), a class A wideband RF power amplifier (1040 L, E&I Ltd., NY, USA) and an impedance matching circuit connected to an ultrasonic transducer (PZT-5A, Piezo Inc., 3.6 × 3.6 × 2 cm^3^). The transmitting face of the PZT transducer was laid with an acoustic matching layer. The ultrasonic power utilized a continuous wave (i.e., sinusoidal wave at the resonant frequency), which allows reflection in the body and significantly reduces the angular alignment sensitivity between the transmitter and the microprobe^23^.

The input ultrasonic intensity was also kept below the approved FDA limit (peak intensity of 720 mW/cm^2^) for the imaging application to avoid cavitation and heat-induced damage^35^. All acoustic intensities were calculated from the measured peak acoustic pressure by a fiber optic hydrophone (FOH, Precision Acoustics, UK). The measured voltage output, *V*_*FOH*_ (mV), from the hydrophone (monitored using an oscilloscope) was converted to peak acoustic pressure, *P*_*peak*_ (MPa), by the following equation.

$${P}_{peak}=\frac{{V}_{FOH}}{Sensitivity\,}$$

The calibrated sensitivity of the fiber optic hydrophone was 215.94 mV/MPa. The peak acoustic pressure was then converted to the acoustic intensity, *I* (mW/cm^2^), by the following equation:

$$I=\frac{{P}_{peak}^{2}}{2\,{Z}_{water}}$$

The *Z*_*water*_ is a specific acoustic impedance (=1.43 MRayl), which is defined as *Z=ρν*_*Water*_, where *ρ* is the density (kg/m^2^) and *ν*_*Water*_ is the speed of sound in water (m/s). Note that the prefactor 1/10 can be added to display the acoustic intensity in mW/cm^2^ (1 W/m^2^ = 10 mW/cm^2^).

### Ablation zone estimation

Figure 3a shows an experimental setup for the ablation zone estimation. We used a 0.7% (w/v) agarose gel with a pH indicator (i.e., phenol red) for visualization of the ablation zone. Agarose gel was used since it is electrically neutral and mechanically stable at room temperature; this gel is a well-established phantom to assess EA^2,36,37^. The pH in the agarose gel was adjusted to a physiological pH of 7.4 by adding 0.9 wt/% sodium chloride. Then, we added 1 ml of phenol red per 100 ml of agarose solution for colorimetric monitoring of pH changes. Phenol red dye turns yellow in an acidic environment and red in an alkaline environment. A central crater was carved out in the agarose gel and filled with deionized water. The microprobe was then placed in the crater, and the electrodes were inserted into the agarose gel. Time-lapse images were captured for visual tracking of pH fronts during the experiment (images are captured at intervals of 5 min over an hour). The images were then processed and analyzed using ImageJ software (NIH) to measure the area of color-changed regions of the agarose gel.

### Cells and cell viability staining

Figure 4a shows an experimental setup for the *in vitro* cell viability test. We separated the microprobe operation and electrolysis for experimental convenience. This approach also ruled out an ultrasound effect in the EA. HMT3522 S1 cells were obtained from Sigma. HMT3522 cells were cultured in DMEM supplemented with glutamine, penicillin, streptomycin, and 10% FBS (Sigma). We first eliminated the possibility of temperature and undesired pH changes using 25 mM HEPES buffer in PBS. After the microprobe operation, we used the Trypan Blue cell viability assay. After collecting supernatant cells, adherent cells were released by 6 min 0.25% Trypsin-EDTA. All dead cells were then stained with 0.4% Trypan Blue. After centrifuging and washing the pellets with PBS twice, the pellets were resuspended in a new plate. We then counted the number of stained cells, corresponding to cell death.

### *Ex vivo* experiments and immunofluorescence/immunohistochemistry

All experiments were approved and conducted according to the regulations and guidelines of the Gangnam Severance Hospital Institutional Animal Care and Use Committee and Institutional Ethics Committee. Livers were harvested and kept in 4 °C HBSS during the experiment. After the microprobes were inserted into the liver (Fig. 4b), ultrasonication was performed for 30 min. Note that the distance between electrodes was 1 cm. After the microprobe operation, livers were washed with PBS and fixed in 4% PFA O/N. The paraffin sections were deparaffinized in xylene and rehydrated using graded ethanol. Antigen retrieval was performed using unmasking solution (Vector Laboratories) in a humidified chamber. Samples were blocked with 5% BSA and 5% sheep serum in PBST for 1 hour at room temperature. The anti-caspase3 antibody was incubated overnight at 4 °C (AB3623, Millipore, USA). After washing three times with PBST, Alexa 594 secondary antibodies were applied for 30 min at room temperature. The samples were then washed again and mounted with Prolong Gold antifade reagent with DAPI (Jackson). Images were taken using an Olympus BX43. For immunohistochemistry, HRP-conjugated secondary antibody was developed using DAB.

### Statistical analysis

The results from independent experiments were expressed as mean ± SD (standard deviation). Statistical analysis of all the experimental data was performed using the Student’s *t*-test with JMP^®^ Pro 14.0. All the experiments were repeated at least quadruple. Data were considered statistically significant when *P*-value is less than 0.05.

## Supplementary information

Supplementary Information.
