# Supplementary material for: An Ultrasonically Powered Implantable Microprobe for Electrolytic Ablation
Source: Sci Rep. 2020 Jan 30;10:1510. doi: 10.1038/s41598-020-58090-8 (PMC6992771; doi:10.1038/s41598-020-58090-8)
Supplement: Supplementary file 1 — Supplementary Information. [file 41598_2020_58090_MOESM1_ESM.pdf]

An Ultrasonically Powered Implantable Microprobe for Electrolytic Ablation

A. Kim[a*], SK. Lee[b], T. Parapudi[c,d], R. Rahimi[c,d], S. H. Song[e], M. C. Park[a], S. Islam[a], J. Zhou[c,d], A. K. Majumdar[a], J. S. Park[f], J.M. Yoo[g], and B. Ziaie[c,d*]

[a]*Department of Electrical and Computer Engineering, Temple University, Philadelphia, PA 19122 USA*
[b]*Jubilee Biotechnology LLC., Philadelphia, PA 19122 USA*
[c]*School of Electrical and Computer Engineering, Purdue University, West Lafayette, IN 47907 USA*
[d]*Birck Nanotechnology Center, West Lafayette, IN 47907 USA*
[e]*Department of Electronic Engineering, Sookmyung Women's University, Seoul, Republic of Korea*
[f]*Pancreatobiliary Cancer Clinic, Department of Surgery, College of Medicine, Gangnam Severance Hospital, Yonsei University, South Korea*
[g]*Department of Microbiology, School of Medicine, CHA University, Seongnam, South Korea*

*Co-corresponding authors

Email addresses: albertkim@temple.edu (Albert Kim) and bziaie@purdue.edu (Babak Ziaie)

Supplementary Figure

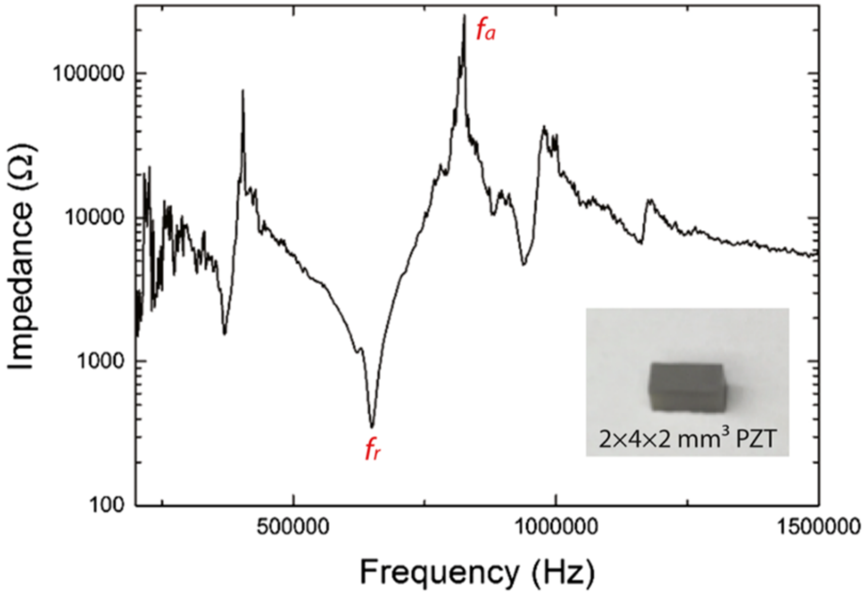

Supplementary Fig. 1 | Impedance over various frequency: measured resonant frequency (the frequency of the lowest impedance) of 2×4×2 mm^3 receivers was 650 kHz

Supplementary Information

In Figure 1c, the solid line box represents an electric port and dashed line box represents a mechanical port. The electrical to mechanical conversion is proportional to the piezoelectric (or electromechanical) coefficient. The piezoelectric coefficient is represented by a transformer (1:ϕ). In the transmitter, the conversion of the electrical signal to ultrasonic waves occurs at the center shim of the transducer. From the center of the transducer, ultrasonic waves propagate towards both the front and back faces of the transducer. The acoustic impedances on pathways from the center to both faces are modeled with transmission lines ($Z_{transmitter}$ or $Z_{receiver}$). The back face of the transmitter is air-backed (modeled as a short circuit); thus, the ultrasonic waves reflect toward the front face. These ultrasonic waves then travel through the body. The tissue attenuation is $\alpha = e^{-\mu(\omega)x}$, where ω is the angular frequency, μ is the attenuation coefficient ($\mu(\omega) \sim \omega^2$), and x is the implantation depth. Therefore, the overall ultrasonic waves that reach the microprobe are reduced and modeled as αT, where T is the transmitted ultrasonic waves. However, this attenuation is negligible, and the transmitted power is expected to be > 90% [28,47]. Although, the attenuation should be considered in the future human use. Higher power transfer efficiency even with attenuation is due to the reflections of ultrasonic waves at the body/air interfaces due to the large acoustic impedance mismatch [24]. The ultrasonic waves reflect back to the body (and microprobe) and allow for an increased wireless power transfer efficiency.

The receiver of microprobe is the same piezoelectric material in smaller form factor (2 × 4 × 2 mm^3). Such small receiver can convert the incoming ultrasonic waves back to the electrical energy. In the electrical port of the receiver, the voltage source (V_{out}) is subject to a capacitor due to paralleled nickel plates of for electrical connection and frequency dependent acoustic reactance (X_R), which becomes shorted if the operation is at the resonant frequency. Therefore, the receiver model can be simplified as an AC source with a source capacitor (C_R). The converted electrical power is then applied across the load (i.e., electrodes) through a rectifier.
